# Subcortical Volume Changes in Early Menopausal Women and Correlation With Neuropsychological Tests

**DOI:** 10.3389/fnagi.2021.738679

**Published:** 2021-12-08

**Authors:** Si Zhang, Weijie Fan, Hao Hu, Li Wen, Mingfu Gong, Bo Liu, Junhao Hu, Guanghui Li, Dong Zhang

**Affiliations:** Department of Radiology, XinQiao Hosptial, Third Military Medical University, Chongqing, China

**Keywords:** early menopausal women, FreeSurfer, subcortical and cortical volume, amygdala, cognition, emotion

## Abstract

**Background:** The aging process and declining estradiol levels are two important factors that cause structural brain alterations. Many prior studies have investigated these two elements and revealed controversial results in menopausal women. Here, a cross-sectional study was designed to individually evaluate estradiol-related structural changes in the brain.

**Methods:** A total of 45 early menopausal women and 54 age-matched premenopausal controls were enrolled and subjected to magnetic resonance imaging (MRI) scans, blood biochemistry tests, and neuropsychological tests. MRI structural images were analyzed using FreeSurfer to detect changes in subcortical and cortical volumes as well as cortical thickness. Finally, structural brain data as well as clinical and neuropsychological data were used for Pearson’s correlation analyses to individually determine estradiol-related structural and functional changes in the brains of early menopausal women.

**Results:** Compared with the premenopausal controls, the early menopausal women showed significant subcortical volumetric loss in the left amygdala and right amygdala, higher serum follicle-stimulating hormone (FSH) levels, more recognizable climacteric and depressive symptoms, decreased quality of sleep, and decreased working memory and executive functions. Simultaneously, FSH levels were related to lower working memory accuracy and longer working memory reaction time. Decreased subcortical volume in the bilateral amygdala was also related to lower working memory accuracy and longer executive reaction time in early menopausal women.

**Conclusion:** The data suggest that estradiol deficiency in early menopausal women can lead to subcortical volume and functional brain changes, which may contribute to further understanding the neurobiological role of declined estradiol levels in early menopausal women.

## Introduction

With the aging process and a progressive reduction in naturally circulating levels of sex hormones, menopausal women experience a range of menopausal symptoms, including declined cognition and structural alterations in the brain (Engler-Chiurazzi et al., 2016; Koothirezhi and Ranganathan, 2021; Peacock and Ketvertis, 2021). To illuminate the neuroprotective affection of estradiol, magnetic resonance imaging (MRI) studies have been performed, but paradoxical outcomes have emerged.

Some studies found that postmenopausal women who received hormone therapy (HT) displayed larger hippocampal volumes and better cognition than those who used placebo (Boccardi et al., 2006; Genazzani et al., 2007; Erickson et al., 2010a). In contrast, other studies demonstrated no differences in hippocampal volume and cognition before and after HT (Eberling et al., 2004; Raz et al., 2004). In addition, a handful of studies revealed that hippocampal and prefrontal cortex volumes were smaller in women who were subject to HT than in controls (Coker et al., 2009). It should be noted that these studies combined the two factors of sex hormone deficiency and age, which worked together to cause the neurotoxicity effects (Jose et al., 2013; Grimm and Eckert, 2017). There have been very few neuroimaging studies that individually evaluated hormone-related changes in the brain among perimenopausal women.

Previous research found obvious estradiol-related alterations in cerebral activity in the left amygdala and bilateral middle occipital gyrus in early menopausal women. However, no significant voxel-based morphometry structure changes were found (Zhang et al., 2018). It is noted that these findings differ from a common perspective that postmenopausal women who receive HT, who exhibit higher serum estradiol levels, show larger hippocampal/parahippocampal volumes than those receiving placebos (Morrison et al., 2006; Lord et al., 2008; Girard et al., 2017). It is suspected that the possible reason for these results was that the experimental subjects were in a state of sex hormone deficiency, which contrasts to subjects in prior research.

Current mainstream opinions in neuroscience consider the view that the hippocampus/parahippocampus-related brain function changes in menopausal women compared to healthy controls (Lord et al., 2008; Rebekah and Christian, 2013; Miller and Harman, 2017). Thus, it was proposed that there may be volumetric changes in the substructures that make up the hippocampus/parahippocampus instead of the hippocampus/parahippocampus in early menopausal women. However, there is no relevant research to support this hypothesis. In addition, to the best of our knowledge, cognitive function is related to cortical thickness and cortical volume, which has also been demonstrated by MRI studies of healthy and brain-lesioned subjects (Watson et al., 2015). Thus, novel techniques should be sensitive enough for segmentation of the subcortical structure of the hippocampus or parahippocampus, which may reveal differences between early menopausal women and controls (Albert et al., 2017).

The FreeSurfer automated segmentation procedure is a novel and reliable method to measure hippocampal subregional volumes (Schmidt et al., 2018; Chiappiniello et al., 2021; Figueiredo et al., 2021). It has been authenticated in many studies, including those investigating Alzheimer’s disease (Novak and Einstein, 2013), schizophrenia (Tost et al., 2013), and fetal alcohol spectrum disorder (Guio et al., 2016). In the present investigation (Zhang et al., 2018), 45 early menopausal women with naturally low endogenous estradiol levels were enrolled. A total of 54 age-matched premenopausal women with naturally low endogenous estradiol levels were also enrolled. A cross-sectional survey that aimed to eliminate the influences of age-related structural alterations in the brain was designed to investigate the modulatory effects of hormones on subcortical brain regions using a neuroimaging approach. Subsequently, subcortical volumes as well as clinical and neuropsychological data were used for Pearson’s correlation analyses to individually determine the early menopausal women’s hormone-related structural and functional brain changes. The goal was to illuminate subcortical structural and volume changes in the brains of early menopausal women and their relation to neurocognitive function.

## Materials and Methods

### Participants

The local Medical Research Ethics Committee of Xinqiao Hospital (Chongqing, China) approved this study protocol, and all subjects provided written, informed consent. A portion of the participants were recruited through advertisements in the community, and others were recommended by gynecology clinics. A total of 45 right-handed early menopausal women (age range: 45–51 years, mean age: 47.38 ± 1.65 years) who fulfilled the following criteria were enrolled (Chen et al., 2017): (1) irregular menstrual cycle length (i.e., menstrual disorders); and (2) 2 or more of the last 10 menstrual cycles showed an adjacent menstrual cycle length change of at least 7 days (Menopause Subgroup et al., 2018). A total of 54 right-handed and education-matched premenopausal control women (age range: 45–49 years, mean age: 46.89 ± 1.69 years) with regular menstrual cycles were also enrolled.

All participants were excluded before enrollment for the following conditions: estradiol or progestational hormone use in the past 6 months, bilateral oophorectomy, Schizophrenic disorder, mood (emotion) disorder, personality and behavior disorders, mental developmental disorders, no specific mental disorders, drug or alcohol abuse, history of traumatic brain injury, neuroanatomical abnormalities in the brain, and chronic diseases (i.e., diabetes, hypertension) that may affect brain structure (Liu et al., 2017). Finally, participants with MRI contraindications and poor image quality were excluded (Bixo et al., 2018).

### Experimental Design

To eliminate possible influences caused by the aging process, our team strictly selected women in the age range of 45–50 years old so that the specific effects of estradiol on brain structure could be determined. All members tested participants’ serum follicle-stimulating hormone (FSH) and estradiol (E2) levels, clinical symptoms as measured by the specialized Kupperman Index (KI, which describes common symptoms and weighs these factors to evaluate the severity of climacteric symptoms), and scores on the Pittsburgh Sleep Quality Index (PSQI). Other measures that were taken included a neuropsychological assessment using the Beck Depression Inventory II (BDI- II, self-reported depression levels), SAS (self-rating anxiety scale) and MRI scans. Finally, cognitive tests were carried out by the E-prime program on the computer, and executive function and working memory were the main focus of the cognitive domains. Executive function was evaluated by the Stroop Test. The Stroop test were conducted as follows: firstly, the task had four types of stimuli, namely four types of color and words with “red,” “yellow,” “green,” and “blue”; secondly, the subjects ignored the word meaning and only responded to the color; at last, we calculate the reaction time under inconsistent conditions and the reaction time under consistent conditions. The ability of memory was evaluated by the Two-back working memory task. While undergoing all of these assessments, the premenopausal controls were required to be in the follicular phase of the menstrual cycle.

### Image Acquisition

Participants underwent MRI scans using a 3.0 T GE MRI system. Acquisition included high-resolution T1-weighted images using a three-dimensional, fast spoiled, gradient-echo (3DSPGR) sequence with a standard eight-channel phased-array head coil and approximately matched parameters (31.25 Hz/pixel bandwidth, TE = 2.8 ms, TR = 450 ms, flip angle = 15°). All scans were 3D sagittal acquisitions with 124 contiguous slices (slice thickness = 1.6 mm, slice gap = 0 mm; FOV = 240 mm × 240 mm, matrix = 256 × 256, isotropic voxel size = 1.6 mm × 1.6 mm × 1.6 mm). Acquisition time was 4 min plus 19 s. Subjects were required to make no movements and close their eyes during the scanning course (Liu et al., 2020).

### Standard FreeSurfer Processing Pipeline

For analysis, the T1-weighted images with indistinct gray-white demarcation and obvious motion artifacts were excluded first. The cerebrum cortical reconstruction and volumetric segmentation were conducted by the FreeSurfer image analysis suite (version 6.0), which has good test-retest reliability on scanners of different manufacturers (Chiappiniello et al., 2021). Field strengths were freely obtained online,^1^ and technical details of these procedures are described in prior publications.

The processing steps were as follows: (1) motion correction; (2) separation of the brain and non-brain tissue using a hybrid watershed or surface deformation procedure; (3) automated Talairach transformation; (4) segmentation of subcortical white matter and deep gray matter brain structures (i.e., hippocampus, amygdala, thalamus, caudate, putamen, pallidum, and ventricles); (5) intensity normalization; (6) tessellation of the boundaries of the gray and white matter, (7) topology correction, (8) surface deformation; and (9) use of the intensity gradients to properly divide the gray/white and gray/cerebrospinal fluid boundary, which defined the greatest shift in intensity in the other tissue (Brown et al., 2020).

Once the standard processing stream was completed, the data was inspected, and accuracy of the boundaries between gray/white and gray/cerebrospinal fluid were confirmed. Some subjects needed minimal edits, in which case images were run through the second reconstruction, beginning at the point where the edits were applied.

### Statistical Analysis

Two-sample *t*-tests were conducted with the statistical software program SPSS 22.0 to detect group differences in subcortical volume, demographic characteristics, clinical data, neuropsychological results, and accuracy and reaction times of the Stroop task and Two-back task. Statistical inspection level was defined as *p* = 0.05. Simultaneously, differences in subcortical volume data were controlled for multiple comparisons using the standard false discovery rate (FDR) approach with a false-positive rate of 5% (*p* = 0.05).

To investigate putative diagnostic-specific relationships and acquire partial correlation coefficients for further group comparisons, hierarchical regression analyses were performed for subcortical volume, clinical data, and neurocognitive function variables. Age and educational degrees were regarded as the covariates. Next, Pearson’s correlation global analyses were conducted with SPSS software, and the statistical significance threshold was set as *p* < 0.05.

## Results

### Demographic, Clinical, and Neuropsychological Results

Demographic, clinical, and neuropsychological group comparisons are exhibited in Table 1. There were no statistically significant differences in age (*p* = 0.151), educational level (*p* = 0.757), SAS (*p* = 0.936) and Stroop accuracy rating (*p* = 0.051). Compared with premenopausal controls, early menopausal women showed prominently higher scores on the KI (*p* = 0.031), BDI- II (*p* < 0.001), and PSQI (*p* = 0.003). Neuropsychological results demonstrated that early menopausal women exhibited longer Stroop test reaction time (*p* < 0.001) and Two-back working memory reaction time (*p* = 0.002) but lower accuracy rate in the Two-back working memory test (*p* < 0.001). Early menopausal women group had obviously higher serum FSH levels (*p* < 0.001) and lower estradiol levels than control group. Hormone levels were too low to be measured accurately in part of the early menopausal women; hence, there were no statistical results for this cohort.

**TABLE 1:** Demographic, clinical, and neuropsychological data.

Characteristics	Early menopausal women	Premenopausal controls	*p*-value
n	45	54	
Age at scan (years)	47.38 ± 1.65	46.89 ± 1.69	0.151 (N.S.)
Years of education	12.93 ± 2.76	13.11 ± 2.90	0.757 (N.S.)
FSH (mIU/ml)	46.69 ± 25.65	13.07 ± 10.18	0.000
Kupperman Index	11.73 ± 7.15	8.80 ± 6.21	0.031
BDI-II	10.91 ± 7.62	5.76 ± 5.39	0.000
SAS	41.53 ± 9.81	41.67 ± 6.44	0.936
PSQI	7.00 ± 3.23	4.91 ± 3.49	0.003
**Two-back**			
ACC	0.9429 ± 0.0277	0.9646 ± 0.0206	0.000
RT (ms)	1113.3818 ± 276.8734	964.3576 ± 192.2480	0.002
**Stroop test**			
ACC	0.8931 ± 0.1037	0.9320 ± 0.0919	0.051 (N.S.)
RT (ms)	831.2380 ± 125.3691	754.6167 ± 84.2718	0.000

*FSH, follicle-stimulating hormone; BDI-II, Beck Depression Inventory II; SAS, self-rating anxiety scale; PSQI, Pittsburgh Sleep Quality Index; ACC, accuracy rating; RT, reaction time; N.S., not significant.*

### Group Differences in Subcortical Volume

A total of 41 selected brain regions were, respectively, segmented. Differences were calculated across the groups, which exhibited that early menopausal women had significant smaller subcortical volumes in the left amygdala (*p* < 0.001), right hippocampus (*p* = 0.045), and right amygdala (*p* = 0.001) (Table 2). After the FDR correction, only the left amygdala and right amygdala reflected these differences. Subsequently, subcortical volume of the two groups in the left amygdala and right amygdala were conducted with hierarchical regression analysis for further Pearson’s correlation and global analysis.

**TABLE 2:** Group differences in subcortical volume.

Brain structure (mm^3^)	Early menopausal women	Premenopausal controls	*p*-value
Left lateral ventricle	6252.21 ± 3503.31	5520.95 ± 2401.37	0.223(N.S.)
Left inf lateral ventricle	163.66 ± 110.36	153.91 ± 129.07	0.691(N.S.)
Left cerebellum WM	15288.99 ± 3624.12	16296.78 ± 3154.71	0.142(N.S.)
Left cerebellum cortex	44147.05 ± 3594.56	44710.40 ± 4449.10	0.496(N.S.)
Left thalamus proper	7463.40 ± 884.33	7564.60 ± 752.37	0.540(N.S.)
Left caudate	3223.36 ± 360.89	3267.29 ± 423.03	0.584(N.S.)
Left putamen	4774.27 ± 619.40	4921.89 ± 654.90	0.255(N.S.)
Left pallidum	1166.98 ± 192.86	1159.55 ± 244.04	0.869(N.S.)
3rd Ventricle	899.43 ± 239.45	866.91 ± 213.48	0.477(N.S.)
4th Ventricle	1506.30 ± 327.59	1578.42 ± 410.91	0.344(N.S.)
Brainstem	18776.61 ± 1463.83	18770.68 ± 1786.76	0.986(N.S.)
Left hippocampus	4264.5 ± 378.73	4388.35 ± 325.86	0.083(N.S.)
Left amygdala	1456.82 ± 161.14	1659.83 ± 184.13	0.000
CSF	1064.46 ± 171.35	1053.93 ± 186.33	0.772(N.S.)
Left accumbens area	506.14 ± 86.70	509.22 ± 87.17	0.861(N.S.)
Left ventral DC	3918.08 ± 429.21	4005.81 ± 498.07	0.355(N.S.)
Left vessel	76.63 ± 27.61	87.97 ± 34.66	0.079(N.S.)
Left choroid plexus	1046.57 ± 251.98	1071.09 ± 188.52	0.581(N.S.)
Right lateral ventricle	5294.26 ± 2777.51	5089.72 ± 2360.91	0.693(N.S.)
Right inf lateral ventricle	176.00 ± 135.55	134.56 ± 88.30	0.070(N.S.)
Right cerebellum WM	16407.57 ± 4514.47	16265.99 ± 4161.08	0.871(N.S.)
Right cerebellum cortex	45685.92 ± 4384.58	45987.90 ± 4765.02	0.746(N.S.)
Right thalamus proper	6581.79 ± 535.33	6735.46 ± 564.35	0.171(N.S.)
Right caudate	3248.55 ± 334.75	3219.34 ± 344.27	0.671(N.S.)
Right putamen	4443.09 ± 527.18	4600.41 ± 541.89	0.149(N.S.)
Right pallidum	1184.70 ± 239.19	1204.79 ± 209.65	0.657(N.S.)
Right hippocampus	4317.52 ± 283.55	4449.22 ± 349.49	0.051(N.S.)
Right amygdala	1556.95 ± 135.78	1671.55 ± 182.06	0.001
Right accumbens area	471.36 ± 78.36	495.97 ± 80.29	0.128(N.S.)
Right ventral DC	4054.47 ± 461.67	4142.84 ± 485.22	0.359(N.S.)
Right vessel	83.15 ± 31.99	88.62 ± 47.44	0.512(N.S.)
Right choroid plexus	1135.38 ± 357.74	1198.40 ± 220.87	0.286(N.S.)
Optic chiasm	288.21 ± 59.41	295.87 ± 57.85	0.518(N.S.)
CC_posterior	906.81 ± 153.71	915.53 ± 114.45	0.747(N.S.)
CC_mid-posterior	431.92 ± 98.07	435.34 ± 104.08	0.868(N.S.)
CC_central	502.99 ± 138.19	517.19 ± 120.47	0.586(N.S.)
CC_mid-anterior	459.74 ± 118.25	469.97 ± 97.08	0.637(N.S.)
CC_anterior	746.52 ± 120.49	787.42 ± 116.37	0.090(N.S.)
lhSurfaceHoles	60.77 ± 17.26	58.90 ± 12.54	0.535(N.S.)
rhSurfaceHoles	62.24 ± 12.29	64.75 ± 17.18	0.413(N.S.)
SurfaceHoles	123.02 ± 27.31	123.66 ± 26.50	0.906(N.S.)

*CSF, cerebrospinal fluid; WM, white matter; inf, inferior; lat, lateral; DC, dorsal cortex; CC, cerebral cortex; lh, left hemisphere; rh, right hemisphere; N.S., not significant.*

### Correlations Among Subcortical Volume, Clinical Data, and Neuropsychological Results

**FIGURE 1:**
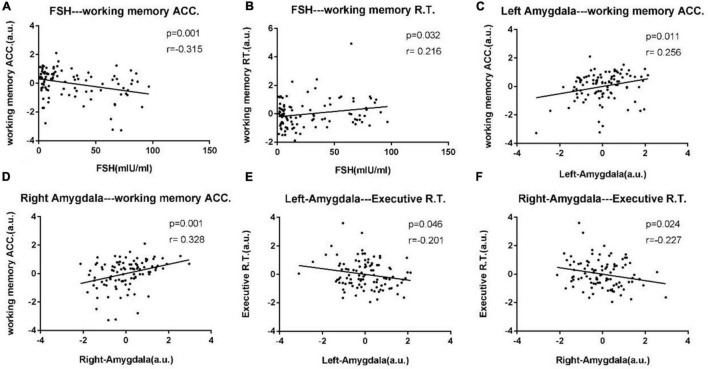
Scatter diagrams exhibit significant pair-wise correlations among bilateral amygdala, clinical data, and neuropsychological data from all experimental participants in **(A–F)**. **(A)** Increased serum FSH levels were negatively correlated with working memory accuracy (*p* = 0.001, *r* = –0.315). **(B)** Increased serum FSH levels were positively correlated with working memory RT (*p* = 0.032, *r* = 0.216). **(C,D)** The Two-back working memory accuracy rate was positively correlated with left amygdala (*p* = 0.011, *r* = 0.256) and right amygdala (*p* = 0.001, *r* = 0.328). **(E,F)** Executive reaction time was negatively correlated with left amygdala (*p* = 0.046, *r* = –0.201), right amygdala (*p* = 0.024, *r* = –0.227).

After correcting for age and educational level, Pearson’s correlation analyses were performed for all experimental data. Results showed that the increased serum FSH levels were negatively correlated with working memory accuracy (*p* = 0.001, *r* = –0.315) and positively correlated with working memory RT (*p* = 0.032, *r* = 0.216). The Two-back working memory accuracy rate was positively correlated with left amygdala (*p* = 0.011, *r* = 0.256) and right amygdala (*p* = 0.001, *r* = 0.328). Executive RT was negatively correlated with left amygdala (*p* = 0.046, *r* = –0.201), and right amygdala (*p* = 0.024, *r* = –0.227). The abovementioned outcomes are shown in Figure 1.

## Discussion

This research intended to explore the relationships between subcortical brain volumes and sex hormones; therefore, matching for age and education in both groups was conducted. Prior study results revealed estradiol-related aberrant cerebral activity in the left amygdala and bilateral middle occipital gyrus but no obvious voxel-based morphometry structure alterations in early menopausal women (Zhang et al., 2018). Our team expanded the subject sample size and used a novel and reliable method to detect hippocampal subregional volume.

When comparing between the selected subcortical and cortical brain structures (estimated from FreeSurfer Version 6.0) across the groups, the most obvious alterations in brain structure were shrinkage of the left amygdala and right amygdala in the early menopausal women in contrast to the premenopausal controls. Simultaneously, early menopausal women displayed higher serum FSH levels, more severe climacteric and depressive symptoms, declined quality of sleep, and decreased memory and executive function. In addition, Pearson’s correlation analysis revealed that increased FSH levels were correlated with working memory. Decreased subcortical volume in the bilateral amygdala was also associated with working memory and executive capacity. These results suggest that the subcortical structural alterations were closely correlated with clinical symptoms in early menopausal women, which may provide direction for future studies on biomarkers and medication guidelines in early menopausal women.

Regarding sex hormones, the early menopausal women showed declines in naturally circulating levels of sex hormones (i.e., estradiol, progesterone) as well as a dysregulation of gonadotropin feedback loops, characterized by increasing levels of serum FSH and luteinizing hormone (LH) (Gillies and Mcarthur, 2010; Merlo et al., 2017). Meanwhile, in previously published reports, many estradiol response subtypes have been found in brain regions correlated with memory and executive capacity (i.e., amygdala, hippocampus, cerebral cortex, and basal forebrain) (Rettberg et al., 2014; Velázquez et al., 2021). Besides, endogenous hormones can induce permanent changes in certain tissue throughout the lifespan (Raz et al., 2004; Erickson et al., 2010b; Herting et al., 2015).

In this study, the early menopausal women had lower estradiol levels in contrast to the controls. The exact level of estradiol in some subjects was too low to be detected; thus, there were no statistical or correlation analyses for this crucial hormone in that cohort, so the value of serum FSH was indirectly used to reflect this condition. Furthermore, increased serum FSH levels were negatively correlated with lower working memory accuracy and positively correlated with longer working memory reaction time. This suggests that memory function may decrease as serum FSH levels increase, which is consistent with previous studies.

The amygdala encompasses several subregions with distinct functional and connectional characteristics in humans (Erickson et al., 2010a). Previous studies have shown that the amygdala is related to either pleasant or unpleasant emotions (i.e., fear, anxiety, and depression) (Barrett et al., 2007; Lanteaume et al., 2007; Williams et al., 2010). Other evidence has proven that the left amygdala is involved in the regulation of memory consolidation by emotional arousal. During this period, synaptic plasticity is promoted by increasing interactions between neocortical storage sites and temporal lobe structures, which involved in declarative memory (Paré et al., 2002; László et al., 2010).

In addition, our prior research on postmenopausal women displayed functional connections between the amygdala and bilateral prefrontal cortex, which may be involved in the neuropathological mechanisms underlying executive function impairments. In this study, decreased subcortical volume in the bilateral amygdala in early menopausal women were associated with prominently higher scores on depression scales, lower accuracy rate in the Two-back working memory test, and longer executive reaction time in an executive function exam. Meanwhile, Pearson’s correlations analyses showed that reduced subcortical volume in the bilateral amygdala was positively associated with lower working memory accuracy and negatively associated with longer executive reaction time. These results are consistent with the corresponding preceding results, which indicate that the amygdala plays a key role in modulating mental and cognitive functions in early menopausal women.

Two limitations in this research should be noted. First, this is a prevalence survey, and whether the alterations in brain structure and function are reversible after estradiol replacement therapy remains to be investigated using another experimental design. Second, no correlation results between sex hormones and the changes in brain structure were found since this experiment was performed in hospital, so the exact level of estradiol was unattainable if the exact value was less than 10. Taking these two limitations into consideration, the exact levels of sex hormones should be tested in a biochemical laboratory. Finally, the early menopausal women who undergo hormone therapy should be added as another control group to confirm whether the changes in brain structure and function are reversed after estradiol replacement therapy in following studies.

## Conclusion

This study, for the first time, clarifies the effects of sex steroid hormones on brain structure in early menopausal women. The study showed that shrinkage in the left amygdala and right amygdala were the most obvious alterations in brain structure. Furthermore, subcortical structural alterations were closely correlated with clinical symptoms in early menopausal women, which may guide the potential use of estradiol for prevention of cognitive impairment and structural brain alterations in menopausal women.

## Data Availability Statement

The raw data supporting the conclusions of this article will be made available by the authors, without undue reservation.

## Ethics Statement

The studies involving human participants were reviewed and approved by the Medical Research Ethics Committee of Xinqiao Hospital (Chongqing, China). The patients/participants provided their written informed consent to participate in this study.

## Author Contributions

DZ, LW, SZ, WF, HH, MG, BL, JH, and GL conceptualized and designed the study. HH, MG, BL, JH, and GL conducted the research. SZ and WF analyzed the data and wrote the manuscript. All authors provided critically revisions to the manuscript.

## Conflict of Interest

The authors declare that the research was conducted in the absence of any commercial or financial relationships that could be construed as a potential conflict of interest.

## Publisher’s Note

All claims expressed in this article are solely those of the authors and do not necessarily represent those of their affiliated organizations, or those of the publisher, the editors and the reviewers. Any product that may be evaluated in this article, or claim that may be made by its manufacturer, is not guaranteed or endorsed by the publisher.

## Footnotes

1
http://surfer.nmr.mgh.harvard.edu
